# Influenza Vaccination Mediates SARS-CoV-2 Spike Protein Peptide-Induced Inflammatory Response via Modification of Histone Acetylation

**DOI:** 10.3390/vaccines12070731

**Published:** 2024-07-01

**Authors:** Zejie Zuo, Yating Mu, Fangfang Qi, Hongyang Zhang, Zhihui Li, Tuo Zhou, Wenhai Guo, Kaihua Guo, Xiquan Hu, Zhibin Yao

**Affiliations:** 1 Department of Rehabilitation Medicine, The Third Affiliated Hospital, Sun Yat-sen University, Guangzhou 510630, China; zuozj3@mail.sysu.edu.cn (Z.Z.);; 2 Department of Neurology, Mayo Clinic, Rochester, MN 55905, USA; 3 Zhongshan School of Medicine, Sun Yat-sen University, Guangzhou 510080, China; 4 Guangzhou Women and Children’s Medical Center, Guangzhou 510620, China; 5 Department of Traditional Chinese Medicine, The Third Affiliated Hospital, Sun Yat-sen University, Guangzhou 510630, China

**Keywords:** COVID-19, vaccine, influenza vaccination, acetylation

## Abstract

The effectiveness of coronavirus disease 2019 (COVID-19) vaccines against the severe acute respiratory syndrome coronavirus 2 (SARS-CoV-2) strain rapidly wanes over time. Growing evidence from epidemiological studies suggests that influenza vaccination is associated with a reduction in the risk of SARS-CoV-2 infection and COVID-19 severity. However, the underlying mechanisms remain elusive. Here, we investigate the cross-reactive immune responses of influenza vaccination to SARS-CoV-2 spike protein peptides based on in vitro study. Our data indicate enhanced activation-induced-marker (AIM) expression on CD4+ T cells in influenza-vaccination (IV)-treated peripheral blood mononuclear cells (PBMCs) upon stimulation with spike-protein-peptide pools. The fractions of other immune cell subtypes, including CD8+ T cells, monocytes, NK cells, and antigen-presenting cells, were not changed between IV-treated and control PBMCs following ex vivo spike-protein-peptide stimulation. However, the classical antiviral (IFN-γ) and anti-inflammatory (IL-1RA) cytokine responses to spike-protein-peptide stimulation were still enhanced in PBMCs from both IV-immunized adult and aged mice. Decreased expression of proinflammatory IL-1β, IL-12p40, and TNF-α is associated with inhibited levels of histone acetylation in PBMCs from IV-treated mice. Remarkably, prior immunity to SARS-CoV-2 does not result in modification of histone acetylation or hemagglutinin-protein-induced cytokine responses. This response is antibody-independent but can be mediated by manipulating the histone acetylation of PBMCs. These data experimentally support that influenza vaccination could induce modification of histone acetylation in immune cells and reveal the existence of potential cross-reactive immunity to SARS-CoV-2 antigens, which may provide insights for the adjuvant of influenza vaccine to limit COVID-19-related inflammatory responses.

## 1. Introduction

The coronavirus disease 2019 (COVID-19) pandemic, caused by severe-acute-respiratory-syndrome-related coronavirus-2 (SARS-CoV-2) infection or reinfection, remains a global public health hazard [1]. Extensive vaccination programs (about 50 vaccines) have been approved to prevent severe SARS-CoV-2 infection and death [2]. However, the effectiveness of vaccine-induced protection rapidly wanes over time mainly due to the decrease in the anti-spike-antigen-antibody levels in circulation or the respiratory tract [3]. Cross-reactive immunomodulatory responses induced by non-SARS-CoV-2 vaccines, including influenza vaccine (IV) and Bacille Calmette–Guérin (BCG) vaccine, have been observed to enhance protection against SARS-CoV-2 infection or vaccination outcomes independent of antibody responses [4,5,6,7,8]. Long-term transcriptomic changes have been observed in peripheral blood mononuclear cells (PBMCs) from influenza-vaccinated individuals, associated with the production of a broad range of cytokines upon SARS-CoV-2 antigen stimulation [9]. Understanding cross-reactive immune responses to the SARS-CoV-2 antigen may provide insights for the development of broad-spectrum COVID-19 vaccines and awareness of unbiased vaccine recommendations. However, the exact mechanism by which certain vaccines induce cross-reactivity to the SARS-CoV-2 antigen remains unclear.

Potential explanations for the protective effects of influenza vaccination (IV) against SARS-CoV-2 outcomes include epigenetic modifications of immune cells [9,10] and the induction of a common antiviral infection response [11]. It has been suggested that IV induces sustained hypoacetylation in myeloid cells at the single-cell level, marked by reduced levels of H3K9ac and H3K27ac. This multicellular epigenetic response to the IV stimulation is partly due to histone modifications that lead to chromatin reorganization of inflammatory genes, which facilitates the production of cytokines and contributes to a sustained cross-protective effect against the Zika and dengue viral infections for at least six months [12]. Therefore, we hypothesize that influenza vaccination would induce histone modification of PBMCs, influencing their cytokine response to SARS-CoV-2 antigen stimulation.

Here, we bring the in vitro evidence for cross-reactivity between SARS-CoV-2 and influenza antigens. Our results demonstrate that CD4+ T cells expressed more activation-induced marker (AIM) when IV-immunized PBMCs were stimulated with SARS-CoV-2 spike protein peptide pools. In the culture supernatants from the same PBMC sample, proinflammatory cytokines IL-1β, tumor necrosis factor (TNF)-α, and IL-12p40 were downregulated, while the expression of antiviral cytokines interferon (IFN)-γ and anti-inflammatory interleukin (IL)-1RA increased. The observed downregulation of proinflammatory cytokine responses in stimulated IV-immunized PBMCs were partly attributed to the decrease in histone acetylation levels, as manipulation of the histone acetylation of PBMCs using the histone deacetylase inhibitor TSA abrogated the changes in these cytokine responses. Crucially, pretreatment with the histone acetyl transferase inhibitor A-485 preserved the similar proinflammatory cytokine responses in hemagglutinin-protein (HA) peptide-stimulated SARS-CoV-2 vaccine (SCV)-immunized PBMCs, indicating that histone acetylation may be an important pattern of epigenomic reprogramming in PBMCs to affect the induction of anti-inflammatory responses following influenza vaccination.

## 2. Materials and Methods

### 2.1. Animals and Immunization

Male-specific pathogen-free C57BL/6 mice aged 6-8 weeks (about 20 g) or 20 months (about 45 g), purchased from Guangdong Medical Laboratory Animal Center, were vaccinated on day (D) 0 with 50 µL of the inactivated quadrivalent influenza vaccine (1.5 µg antigen concentration) (Hualan Bio, included A/Michigan/45/2015 (H1N1) pdm09-like virus, A/Singapore/INFIMH-16-0019/2016 (H3N2)-like virus, B/Colorado/06/2017-like virus, and B/Phuket/3073/2013-like virus), SARS-CoV-2 vaccine (0.65U antigen concentration) (China’s National Biotechnological Group, included 19nCov-CDC-Tan-HB02), or sterilized phosphate buffer saline (PBS) into the left leg (*n* = 6/group). Sera were collected on 5 days after injection (D5) by the tail vein and on D14 by the postorbital vascular plexus under sedation with intraperitoneal (i.p.) injection of tribromoethanol (250 mg/kg) and tert-amyl alcohol (2.5%). All mice were kept in microisolator cages with free access to food pellets and water under a 12 h light/dark cycle and housed in standardized conditions at a specific-pathogen-free (SPF) facility. The animal study protocol was approved by the Ruiye model animal Biotechnology Co., Ltd. (Guangzhou, China) Experimental Animal Ethics Committee Inspection (protocol number RYEth-20230815297).

### 2.2. RBD-Antibody Enzyme-Linked Immunosorbent Assay (ELISA)

To measure the levels of anti-SARS-CoV-2 RBD antibodies in mice, RBD-neutralizing-antibody test kits (Vazyme, Nanjing, China, cat# DD3101) were used according to the manufacturer’s instructions. Briefly, serum samples were diluted 1:10 in dilution buffer, and the optical density (OD) at 405 nm was measured by a plate reader (Sunrise) after incubation. The OD450 measurements of negative controls and samples in a single assay were used to calculate the antibody neutralization titer in the tested serum. Only when the neutralization rate was equal to or greater than 20% did the detected samples contain SARS-CoV-2-neutralizing antibodies.

### 2.3. Hemagglutination-Inhibition (HI) Assay

To measure the levels of influenza-specific antibody in mice, an HI assay was used according to an established procedure and with chicken erythrocytes [13]. Briefly, serum samples were treated with a receptor-destroying enzyme at 37 °C for 18 h and heated at 56 °C for 30 min. They were then diluted in serial two-fold dilutions with PBS. The highest dilution that caused complete hemagglutination inhibition against four hemagglutination units (HAU) of virus was recorded asthe HI titer.

### 2.4. Peptide Pools

SARS-CoV-2 spike glycoprotein peptide pools and HA-peptide pools were purchased from Genscript (Nanjing, China). The spike-glycoprotein-peptide pool includes 316 peptides derived from a peptide scan through the entire spike glycoprotein of SARS-CoV-2. As previously described [14], thirteen influenza A/HA subtypes and B/HA peptides were synthesized to make HA-peptide pools for the next stimulation.

### 2.5. Cell Isolation and In Vitro Stimulation

PBMCs were isolated from heparinized venous blood of immunized mice, followed by Ficoll (Sigma-Aldrich, St. Louis, MO, USA, cat# 1077) density-gradient centrifugation. PBMCs were either used fresh or stored in liquid nitrogen, freezing with media (70% RPMI-1640, 20% FBS, and 10% DMSO) until use. For in vitro stimulation [12], 200 μL of cell suspension (RPMI-1640 culture medium supplemented with 10% FBS, 10 μg/mL gentamicin, 10 mM L-glutamine, and 10 mM pyruvate; total 5 × 10^5^ cells) was added to the well of a 96-well round-bottomed tissue culture plate and mixed with either spike-protein-peptide pools (1 μg/mL, RP30020CN, Genscript) or HA-peptide pools (1 μg/mL, Genscript) in the cultures at 37 °C. After 24 h of incubation, supernatants were harvested and stored at -80 °C for cytokine measurements. Cells were washed with PBS for flow-cytometry experiments. To control the global histone acetylation, trichostatin A (TSA, CST, cat# 9950) or A-485 (Tocris, Bristol, UK, cat# 6387) was added to the media for 2 h of incubation, followed by peptide pool stimulation.

### 2.6. Cytokine Measurements

The expression of IL-1β (Neobioscience, Shenzhen, China, cat# EMC001bQT), TNF-α (Neobioscience, cat# EMC102aQT), IFN-γ (Neobioscience, cat# EMC101gQT), IL-1RA (ELK Biotechnology, Wuhan, China, cat# 1091), and IL-12p40 (ELK Biotechnology, cat# 2507) was measured in the collected supernatants using commercial ELISA kits according to the manufacturer’s instructions. Briefly, samples were diluted in a 1:5 ratio with the provided diluent buffer. After incubation, the OD450 values were measured by a plate reader (Sunrise), and the levels of cytokines were calculated as pg/mL.

### 2.7. Total Histone Extraction and Histone-H3-Acetylation Detection

According to the manufacturer’s instructions, the PBMCs (about 1 × 10^6^ cells) were freshly isolated to extract total histones by using the Total Histone Extraction Kit (EpigenTek, Farmingdale, NY, USA, cat# OP-0006). After that, the H3-acetylation changes were measured using the Total Histone H3 Acetylation Detection Fast Kit (EpigenTek, cat# P-4030). The results were calculated as the percentage of histone-H3 acetylation using the detected OD450 value.

### 2.8. Flow-Cytometry Assays

After ex vivo stimulation, cells were washed with 1 mL of FACS buffer (BD, cat# 554657) and centrifuged at 300× *g* for 5 min at 4 °C. Afterward, the cells were incubated with 200 μL of FACS buffer of antibody cocktail for surface marker staining, including CD3 (ThermoFisher Scientific, Waltham, MA, USA, cat# 12-0038; Biolegend, San Diego, CA, USA, cat# 100233), CD45 (ThermoFisher Scientific, cat# 11-0459), CD4 (ThermoFisher Scientific, cat# 11-0041), CD8 (ThermoFisher Scientific, cat# 25-0088), CD69 (Tonbo, Tucson, AZ, USA, cat# 20-0691), CD137 (ThermoFisher Scientific, cat# 12-1371), CD11b (Tonbo, cat# 20-0112), CD115 (Biolegend, cat# 135527), NK1.1 (ThermoFisher Scientific, cat# 45-5941), and MHC-II (ThermoFisher Scientific, cat# 17-5320). After incubating for 30 min at 4 ℃ in the dark, the cells were washed with 1 mL of FACS buffer. For intracellular cytokine staining, the stimulated PBMCs were additionally incubated for 4 h with brefeldin A (10 mg/mL; Sigma Aldrich, St. Louis, MO, USA, cat# B7651) before surface marker staining. After staining for surface markers, cells were washed and fixed in 1 mL of fixation/permeabilization solution (ThermoFisher Scientific, cat# 88-8824) at 4 °C for 30 min in the dark. Next, 2 mL of permeabilization buffer was added to wash the cells twice, and the cells were centrifuged at 300× *g* for 5 min at 4 °C. Then, the cells were incubated with IL-1β and IFN-γ antibodies (ThermoFisher Scientific, cat# 25-7114 and 12-7311) in permeabilization buffer at 4 °C for 30 min in the dark. Finally, the cells were washed and resuspended in 300 μL of FACS buffer. Data were collected by using a CytoFLEX flow cytometer (Bechman Coulter, Brea, CA, USA) and analyzed using FlowJo 10.8.1 (BD).

### 2.9. Statistical Analysis

GraphPad Prism (v 8.0) and FlowJo (v 10.8.1) were used for all statistical analyses and data visualization. Data are presented as the mean ± SEM, and their normal distribution and variance homogeneity were analyzed first. We used one-way ANOVA followed by Tukey’s post hoc test for multigroup comparisons and used Pearson correlation analysis to examine the associations between the levels of H3 acetylation and the expression of multiple cytokines by PBMCs. A *p* value equal to or less than 0.05 was considered statistically significant.

## 3. Results

### 3.1. Influenza Vaccination Induces Changes in Cytokine Production Capacity in PBMCs after SARS-CoV-2 Spike Stimulation

**Figure 1:**
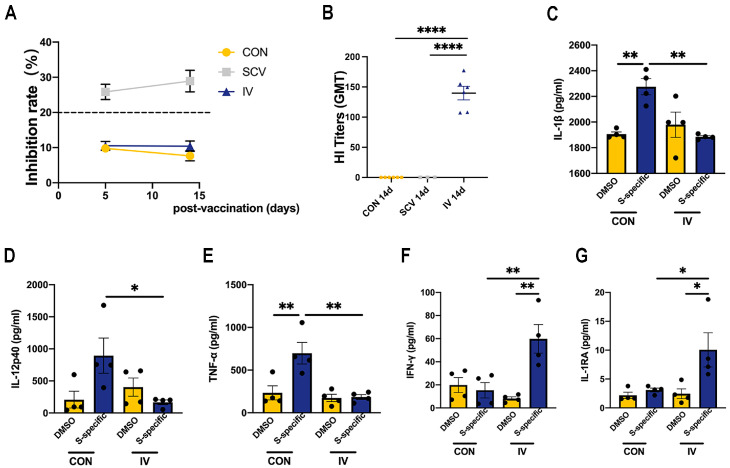
The antibody responses following immunization and cytokine secretion following ex vivo stimulation of PBMCs. Mice (*n* = 3–6/group) were vaccinated intramuscularly with phosphate buffer saline (CON), influenza vaccine (IV), SARS-CoV-2 vaccine (SCV), and blood collected on days 5 and 14. (**A**) SARS-CoV-2 RBD neutralizing antibodies in sera were determined by ELISA and estimated by the inhibition rate with a positive threshold of 20%. (**B**) The geometric mean HI titer (GMT) was detected by HI assay. (**C**–**G**) At 14 days postvaccination, PBMCs were stimulated ex vivo with 1 μg/mL of SARS-CoV-2 spike protein peptide pools, and cytokine production in the culture supernatant was determined by ELISA (IL-1β, IL-12p40, TNF-α, IFN-γ, and IL-1RA) (*n* = 4/group). Data are presented as mean ± SEM and * *p* < 0.05, ** *p* < 0.01, and **** *p* < 0.0001 (one-way ANOVA with Tukey’s test).

We first examined whether vaccine immunization induced the antibody responses in the circulation. As expected, we only observed that the SARS-CoV-2-specific inhibition rate could be maintained up to 20% at 5 days and 14 days after SCV, indicating robust anti-RBD antibody responses in plasma samples (Figure 1A). HI titers were detectable only in the IV group but not in SCV and control groups (Figure 1B). These results suggested a successful immunization.

To detect the functional changes in myeloid cells after influenza vaccination, we isolated PBMCs from vaccinated or control mice and then stimulated them ex vivo with SARS-CoV-2 spike protein peptide pools. Several differentially expressed cytokines were selected and measured in our culture supernatants, as previously reported [9,12]. In agreement, we observed a significant reduction in IL-1β, IL-12p40, and TNF-α expression in IV-immunized PBMCs after stimulation compared to the controls (Figure 1C–E). However, spike-protein-peptide stimulation induced higher IFN-γ and IL-1RA production from IV-immunized PBMCs relative to those of the control (Figure 1F,G). Therefore, these data indicated that IV immunization alters the cytokine production of PBMCs to challenge SARS-CoV-2 antigen stimulation with no association with the SARS-CoV-2-specific antibody response, which is characterized by the manifestation of prominent anti-inflammatory and antiviral cytokine responses.

### 3.2. Influenza Vaccination Enhances Activation-Induced-Marker (AIM) Expression on CD4^+^ T Cells Post SARS-CoV-2 Spike Protein Peptide Pool Stimulation

**Figure 2:**
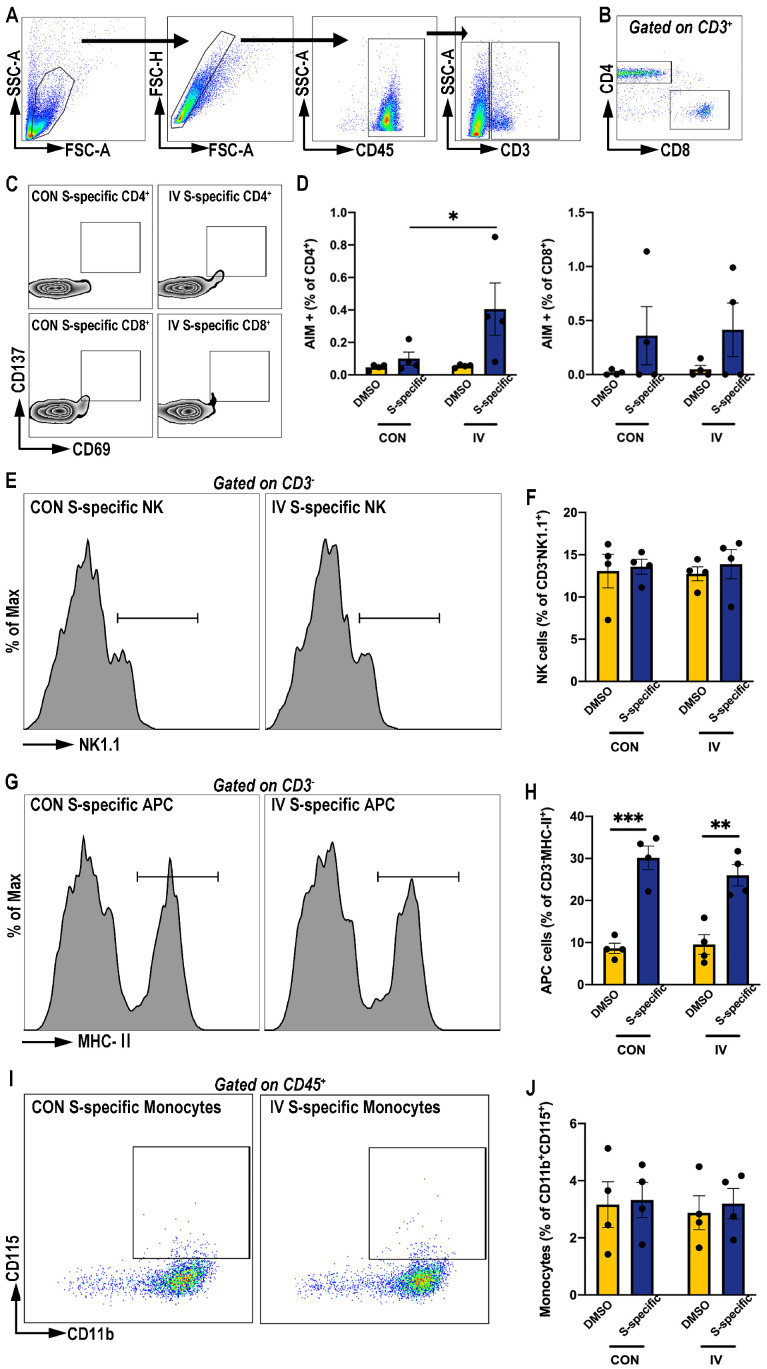
The activation of immune cell subtypes in PBMCs following IV and ex vivo stimulation. (**A**) Representative plots of the gating strategy of CD3^+^ T cells, CD3^-^ immune cells, and CD45^+^ immune cells from PBMCs. (**B**) Representative plots of the T-cell subset gated on total CD3^+^ T cells expressing CD4 and CD8 surface markers. (**C**,**D**) Dot plots of the frequencies of cells gated out of CD4^+^ and CD8^+^ T cells expressing the AIM marker (CD69 and CD137) and the percentage of the designated population (AIM^+^CD4^+^ and AIM^+^CD8^+^) in the IV-immunized or control PBMCs stimulated with SARS-CoV-2 spike protein peptide pools or DMSO. (**E**,**F**) Dot plots of the frequencies of cells gated out of CD3^-^ immune cells expressing the NK1.1 marker and the percentage of the designated population (NK1.1^+^CD3^−^) in the IV-immunized or control PBMCs stimulated with SARS-CoV-2 spike protein peptide pools or DMSO. (**G**,**H**) Dot plots of the frequencies of cells gated out of CD3^−^ immune cells expressing the MHC-II marker and the percentage of the designated population (MHC-II^+^CD3^−^) in the IV-immunized or control PBMCs stimulated with SARS-CoV-2 spike protein peptide pools or DMSO. (**I**,**J**) Dot plots of the frequencies of monocytes gated out of CD45^+^ immune cells expressing CD11b and CD115 markers and the percentage of the designated population (CD11b^+^CD115^+^ CD45^+^) in the IV-immunized or control PBMCs stimulated with SARS-CoV-2 spike protein peptide pools or DMSO. *n* = 4/group. Data are presented as mean ± SEM and * *p* < 0.05, ** *p* < 0.01, and *** *p* < 0.001 (one-way ANOVA with Tukey’s test).

Previous studies have demonstrated that immune cells are the main sources of cytokine expression after in vitro stimulation [12], and T-cell-mediated immunity has been confirmed to provide wide cross-reactivity against SARS-CoV-2 variants [15]. To determine whether IV immunization induced SARS-CoV-2-specific T-cell protective responses, we measured AIM expression within T cells from IV-immunized PBMCs stimulated with SARS-CoV-2 spike protein peptide pools by flow cytometry (gating strategy shown in Figure 2A,B). We observed no significant effects of IV treatment on the AIM^+^ CD8^+^ S-specific T-cell responses (Figure 2C,D). However, IV treatment had a modest but significant effect on S–specific CD4^+^ T cells expressing AIM (CD69^+^CD137+) compared to the control levels (Figure 2C,D).

Additionally, the frequencies of other immune cells in the PBMCs, includeing the natural-killer (NK) cells (CD3^-^ NK1.1^+^) (Figure 2E,F), antigen-presenting cells (CD3^-^ MHC-II^+^) (Figure 2G,H), and monocytes (CD11b^+^ CD115^+^) (Figure 2I,J), were also detected using flow cytometry. However, IV treatment did not change the fractions of these cells in response to spike-protein-peptide pool stimulation. In summary, these data suggest that IV treatment might induce CD4^+^ T cell training and anti-inflammatory responses for the induction of cross-reactive immunity to the SARS-CoV-2 spike protein peptides.

### 3.3. Influenza Vaccination Induces Histone-H3 Hypoacetylation in PBMCs

**Figure 3:**
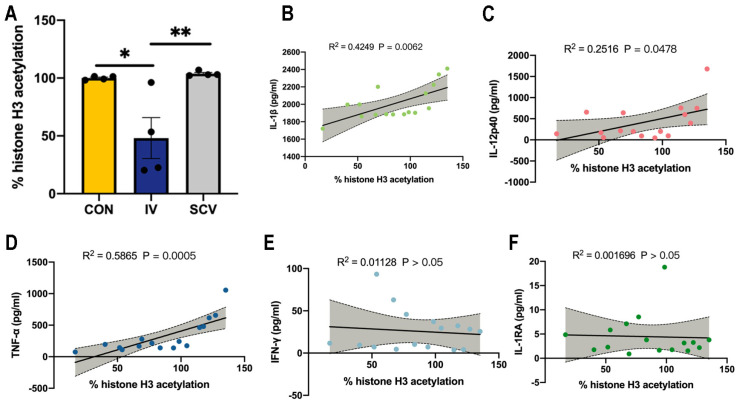
Histone-H3 acetylation changes after IV treatment and correlates with cytokine secretion. (**A**) Histone-H3 acetylation was quantified in PBMCs isolated from the IV, SCV, and control groups by ELISA and was calculated as the percentage of the control histone-H3 acetylation. *n* = 4/group. Data are presented as mean ± SEM and * *p* < 0.05 and ** *p* < 0.01 (one-way ANOVA with Tukey’s test). (**B**–**F**) Pearson correlations of the percentage of histone-H3 acetylation and cytokine secretion after postvaccination PBMCs following ex vivo stimulation. The gray area shows the confidence intervals (95%) (*n* = 4/group).

It has been demonstrated that histone modification levels are related to cytokine production in ex vivo stimulated PBMCs, especially H3 acetylation [12]. We therefore extracted total histones from PBMCs and measured the total histone H3 acetylation. Vaccination with influenza resulted in decreased histone-H3 acetylation in PBMCs compared to those of the controls or SCV immunization (Figure 3A). As expected, the H3-acetylation levels were positively correlated with proinflammatory IL-1β, IL-12p40, and TNF-α production (Figure 3B–D) but were not correlated with IFN-γ and IL-1RA production by PBMCs (Figure 3E,F).

**Figure 4:**
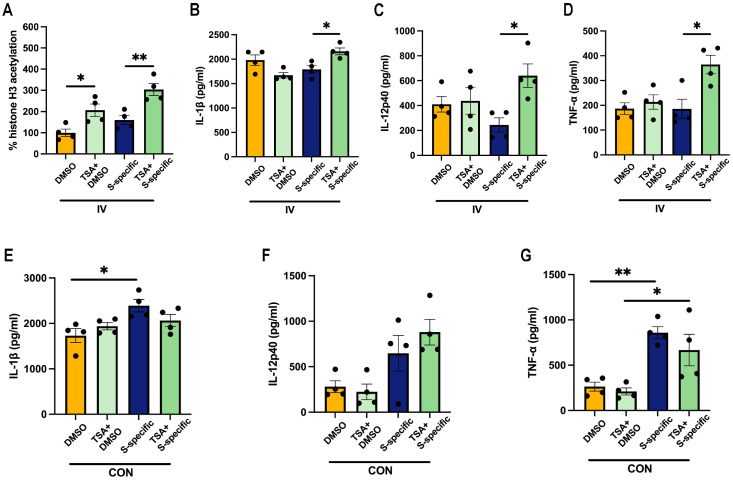
The histone deacetylase inhibitor TSA increased proinflammatory cytokine production by postvaccination PBMCs following ex vivo stimulation. At 14 days postvaccination, PBMCs were pretreated with 50 μL of inhibitor solution containing TSA in complete media and then stimulated ex vivo with 1 μg/mL of SARS-CoV-2 spike protein peptide pools. (**A**) The histone-H3 acetylation was quantified by ELISA and was calculated as the percentage of the DMSO-group histone-H3 acetylation. (**B**–**D**) The production of IL-1β, IL-12p40, and TNF-α in the IV-immunized PBMCs culture supernatant was determined by ELISA. (**E**–**G**) The production of IL-1β, IL-12p40, and TNF-α in the control PBMC culture supernatant was determined by ELISA. *n* = 4/group. Data are presented as mean ± SEM and * *p* < 0.05 and ** *p* < 0.01 (one-way ANOVA with Tukey’s test).

To confirm the role of histone acetylation in proinflammatory cytokine production, we examined whether increased histone acetylation via the deacetylase-inhibitor-TSA treatment affects proinflammatory cytokine production. As expected, TSA treatment led to an increase in histone-H3-acetylation levels in IV immunized-PBMCs following DMSO and spike-protein-peptide pool stimulation (Figure 4A). In line with a previous study [12], PBMCs from IV-immunized mice treated with TSA showed a significant increase in the expression of IL-1β, IL-12p40, and TNF-α in the culture supernatant (Figure 4B–D). Moreover, our results show that the expression of IL-1β, IL-12p40, and TNF-α was unaffected by TAS treatment in the IV-immunized PBMCs in the absence of spike-protein-peptide pool stimulation (Figure 4B–D). We also observed that TSA treatment did not influence the production of IL-1β, IL-12p40, and TNF-α in non-immunized PBMCs (Figure 4E–G). These results suggest that IV immunization may mitigate the proinflammatory cytokine response, at least partially, through the modification of histone-H3 acetylation in PBMCs.

### 3.4. Prior SARS-CoV-2 Vaccination Does Not Rescue Proinflammatory Cytokine Production in HA-Peptide-Pool-Stimulated PBMCs

**Figure 5:**
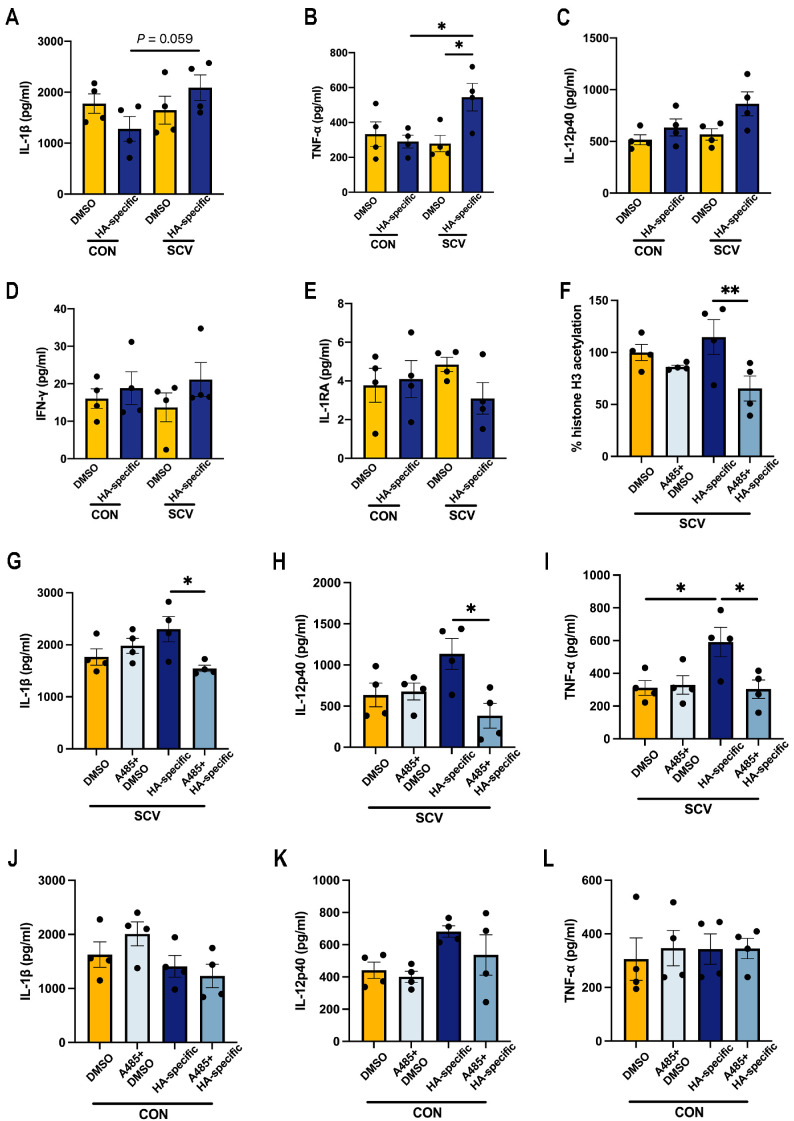
Cytokine secretion following ex vivo stimulation of SCV-immunized PBMCs and the control PBMCs with HA-peptide pools. At 14 days post SCV vaccination or PBS treatment, PBMCs were stimulated ex vivo with 1 μg/mL of HA-peptide pools. (**A**–**E**) Cytokine production in the culture supernatant was determined by ELISA (IL-1β, IL-12p40, TNF-α, IFN-γ, and IL-1RA). PBMCs were pretreated with 50 μL of inhibitor solution containing A485 in complete media and then stimulated ex vivo with HA-peptide pools. (**F**) The histone-H3 acetylation was quantified by ELISA and was calculated as the percentage of the DMSO group histone-H3 acetylation. (**G**–**I**) The production of IL-1β, IL-12p40, and TNF-α in the SCV-immunized PBMCs culture supernatant was determined by ELISA. (**J**–**L**) The production of IL-1β, IL-12p40, and TNF-α in the control PBMCs culture supernatant was determined by ELISA. *n* = 4/group. Data are presented as mean ± SEM and * *p* < 0.05 and ** *p* < 0.01 (one-way ANOVA with Tukey’s test).

To determine whether prior immunity induced by SCV immunization can rescue the cytokine response to influenza, mice were administered SCV i.m. 14 days before their PBMCs were stimulated with hemagglutinin (HA)-peptide pools. This pre-exposure to SCV significantly increased the expression of TNF-α and IL-1β (mildly, *p* = 0.059) in response to HA peptide stimulation (Figure 5A,B). Nonetheless, the expression of IL-12p40, IFN-γ, and IL-1RA in the ex vivo HA peptide-stimulated supernatants from SCV-immunized PBMCs were comparable to those of the control group (Figure 5C–E).

Considering the effect of histone acetylation on cytokine production in PBMCs, we inhibited acetylation by pretreatment with A-485, a histone-acetyltransferase inhibitor. A-485 treatment led to a decrease in histone-H3-acetylation levels in SCV immunized-PBMCs following HA peptide stimulation (Figure 5F). We observed similar expression of IL-1β, IL-12p40, and TNF-α in the A-485-treated cells compared to the unstimulated control (Figure 5G–I). Notably, treatment with A-485 effectively blocked the increase in these proinflammatory cytokines induced by stimulation with SCV and HA peptide (Figure 5G–I). Furthermore, A-485 treatment had no effect on the production of IL-1β, IL-12p40, and TNF-α in PBMCs from non-immunized mice (Figure 5J–L). These data reinforce the concept that histone modification plays a role in modulating cytokine responses in PBMCs in the context of diverse antigen stimulations.

### 3.5. Influenza Vaccination Induces Similar Changes in Cytokine Production Capacity in PBMCs Isolated from Aged Mice

**Figure 6:**
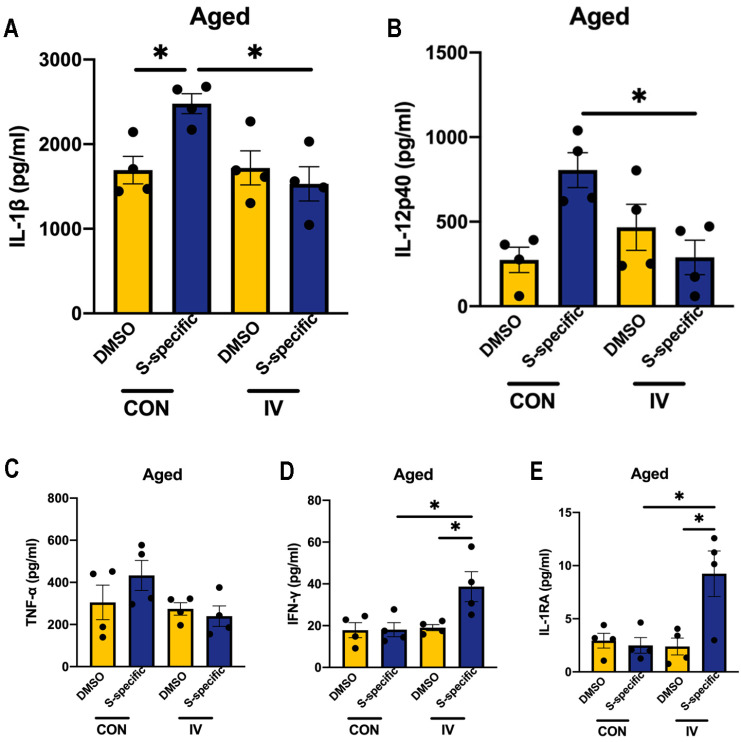
Cytokine secretion following ex vivo stimulation of PBMCs isolated from IV-immunized aged mice with spike-protein-peptide pools. (**A**–**E**) Aged mice (20-month-old) were vaccinated intramuscularly with PBS or IV. At 14 days post-vaccination, PBMCs were stimulated ex vivo with spike-protein-peptide pools, and cytokine production in the culture supernatant was determined by ELISA (IL-1β, IL-12p40, TNF-α, IFN-γ, and IL-1RA). *n* = 4/group. Data are presented as mean ± SEM and * *p* < 0.05 (one-way ANOVA with Tukey’s test).

Immune senescence is common in healthy aging and affects infection pathogenesis and vaccination effectiveness [16]. We examined the spike-protein-peptide-induced expression of the aforementioned cytokines in PBMCs isolated from aged IV-immunized mice (20-month-old). Similar to adult mice, PBMCs isolated from aged IV-immunized mice were also observed to secrete lower levels of IL-1β and IL-12p40, together with higher expression of IFN-γ and IL-1RA after ex vivo spike-protein-peptide stimulation, compared to non-immunized cells (Figure 6). These results further suggested that influenza vaccination may have the similar ability to activate the protected cross-reactive immunity to the SARS-CoV-2 spike antigens in aging.

## 4. Discussion

In this study, we explored the cross-reactive immune responses of influenza vaccination on SARS-CoV-2 spike protein peptides through in vitro experiments. Our findings indicate that prior influenza vaccination induces modification of histone acetylation of PBMCs isolated from both adult and aged mice, which facilitates the suppression of proinflammatory cytokine responses upon exposure to SARS-CoV-2 spike protein peptide.

Influenza vaccinations (IVs) are widely administered to control influenza infection. Given the antigenic drift of influenza strains due to viral mutations, these vaccines are typically administered annually, particularly in young children and the elderly. It has been reported that seasonal influenza vaccination can moderately enhance cross-reactive responses against different influenza viruses, potentially reducing the incidence of infections [17]. Mechanistically, it is well established that influenza vaccination triggers the activation of antigen-specific T lymphocytes and the clonal expansion of B lymphocytes to sustain immune protection [18,19,20,21]. Furthermore, studies have shown that IV administration is linked to a reduced risk of SARS-CoV-2 infection [9,22,23,24,25,26,27,28,29,30]. This protective effect against SARS-CoV-2 has been observed in individuals aged 50 years or older, in which vaccinated individuals exhibited a lower incidence of SARS-CoV-2 infection compared to the unvaccinated counterparts [27,31,32,33,34,35]. These findings support the rationale for IV immunization in eliciting cross-reactive cellular immunity against COVID-19, especially in populations at high risk of infection. Although the exact biological mechanisms remain unclear, the induction of trained immunity by the influenza vaccine is generally accepted [9,12].

Trained immunity is one of the prominent hypotheses that has been highlighted to interpret the beneficial heterologous effects of vaccines [36,37], such as the protective impact of IV or BCG immunization against SARS-CoV-2 infection [9,38]. Previous studies have shown that epigenetic reprogramming of innate immune cells is sufficient to induce trained immunity, which correlate with the antiviral or antibacterial efficacy of vaccines [39]. Wimmers et al. demonstrated that IV immunization induces a persistent change in the epigenome in myeloid cells of humans at the single-cell level, pivotal for shaping trained immunity and modulating responses to unrelated pathogens [12]. Cytokine responses, pivotal in severe disease manifestations, also represent important functional outcomes of trained immunity elicited by vaccination or coronavirus infection [40]. The excessive release of cytokine networks, known as a cytokine storm, significantly contributes to multiorgan pathology and clinical adverse outcomes in pandemic COVID-19 and influenza infection [41,42]. While it is essential for infection clearance, the uncontrolled release of mediators like IL, IFN, and TNF can be detrimental. Vaccination’s key advantage lies in its ability to finely tune immune cell responses to pathogens, facilitating a balanced cytokine response rather than a cytokine storm during infections [43].

An important beneficial effect of vaccination is the induction of a fine-tuning immune cell responses to pathogens, which facilitates a balanced cytokine response rather than a cytokine storm during infection or challenge [43]. IV immunization has been reported to fine-tune pro/anti-inflammatory cytokine production at baseline, potentially rebalancing inflammation and preventing hyperinflammatory responses following SARS-CoV-2 stimulation [9]. Consistent with findings from IV-vaccinated individuals’ PBMCs [9], our study using IV-immunized adult and aged mice demonstrated that increased expression of IL-1β and IL-12p40, achieved by stimulation of PBMCs with spike-protein peptides, can be reduced in culture supernatants from stimulated IV-immunized PBMCs. Conversely, enhanced expression of IFN-γ and IL-1RA was observed post-stimulation, which may help them to induce antiviral and anti-inflammatory responses, thereby contributing to the beneficial anti-SARS-CoV-2 effects of IV immunization.

Cytokine responses represent important functional consequences of epigenetic reprogramming in myeloid cells [44,45]. In agreement with previous studies [12], our findings indicate that the suppression of histone-H3 acetylation in PBMCs following IV immunization functions to influence the production of proinflammatory cytokines in response to spike-protein-peptide stimuli. Previous studies showed that perturbations of histone acetylation affect the secretion of LPS-induced proinflammatory cytokines, such as IL-1β and TNF-α, by monocytes in vitro. This effect is mediated by treating PBMCs with the histone deacetylase inhibitor TSA and the histone-acetyl-transferases-specific inhibitors A-485 [12]. In addition, corticosteroids can downregulate inflammatory gene expression by inhibiting HAT and recruitment of HDAC2. These findings supported and agreed with our observations. Notably, the inhibition of H3K27ac did not eliminate the basal expression of cytokines in cultured PBMCs. Alternative immunologic mechanisms, including heterologous immunity or general antiviral IFN responses, may additionally account for the nonspecific cross-reactivity of the IV immunization and therefore offer protection against unrelated pathogens [27]. However, it has been reported that histone deacetylase can promote SARS-CoV-2 virus entry to the ACE2-expressing cells, especially the lung mesothelial and epithelial cells, and then activate the local inflammatory responses [46,47,48,49]. Thus, the host cells may have heterogeneous effects in response to the different stages of viral infection through histone modifications.

Our data indicate that PBMCs from SCV immunized mice exhibit minimal cross-reactive cytokine responses upon ex vivo stimulation with HA-peptide pools, suggesting a weak or rapidly waning cellular cross-immunity against influenza peptides post-SCV immunization. These observations are supported by the finding from influenza/SARS-CoV-2-coinfected hACE2 mice, where prior immunization with IV, but not SCV, prevented the exacerbation of comorbidity [50]. However, the relevance of SCV immunization on influenza infection warrants further investigation. The clinical prevalence of influenza in individuals vaccinated against SARS-CoV-2 is currently unknown, and it is plausible that SCV immunization may fail to confer protection against influenza due to inadequate immune priming, as multiple SCV immunizations are encouraged to provide an effective defense against COVID-19 [51]. On the other hand, the hACE2 mouse model, which is extensively employed to investigate SARS-CoV-2 pathogenesis [48,49], may exhibit heightened sensitivity to SARS-CoV-2 immunity induction [52,53]. In contrast, the wild-type C57BL/6 mice utilized in our study are an appropriate animal model for influenza or vaccine research.

Our study also has several limitations. For instance, the existence of homologous epitopes between SARS-CoV-2 spike protein peptides and influenza antigens remains undetermined, and such homology could potentially induce a direct T cell cross-reaction [54,55]. Furthermore, our study does not provide direct evidence of the in vivo beneficial effect of IV immunization on hACE2 mice infected with SARS-CoV-2, a gap that requires further investigation.

## 5. Conclusions

We have shown that PBMCs from both adult and aged mice immunized with IV can be activated to induce a protective cross-reactive response to SARS-CoV-2 spike protein peptide stimuli, including the activation of CD4^+^ T cells, the induction of the antiviral cytokine IFN-γ, and a decrease in the levels of IL-1β, IL-12p40, and TNF-α. Mechanistically, acetylation modifications in immune cells following IV immunization may facilitate the cytokine responses. Therefore, periodic recommended vaccination may be a health-promoting behavior likely to serve as a proxy for infectious disease prevention and protection. Further research is warranted to assess the efficacy of other vaccines in eliciting similar protective responses.

## Data Availability

The datasets generated and/or analyzed during this study are available from the corresponding author on reasonable request.
